# Feeding Behaviour and Bioavailability of Essential Amino Acids in Shrimp *Penaeus monodon* Fed Fresh and Leached Fishmeal and Fishmeal-Free Diets

**DOI:** 10.3390/ani11030847

**Published:** 2021-03-17

**Authors:** Cedric J. Simon, Ha Truong, Natalie Habilay, Barney Hines

**Affiliations:** 1 The Commonwealth Scientific and Industrial Research Organisation (CSIRO), Queensland Bioscience Precinct, 306 Carmody Road, St Lucia 4067, Australia; Barney.hines@csiro.au; 2 The Commonwealth Scientific and Industrial Research Organisation (CSIRO), Bribie Island Research Centre, 144 North Street, Woorim 4507, Australia; ha.truong@csiro.au (H.T.); Natalie.Habilay@csiro.au (N.H.)

**Keywords:** feed intake, appetite, crystalline amino acid, leaching, feed frequency

## Abstract

**Simple Summary:**

While success has been achieved with replacing fishmeal entirely in diets for whiteleg shrimp *Litopenaeus vannamei*, giant tiger prawn *Penaeus monodon* still requires fishmeal for optimum culture performance. Crystalline amino acid (CAA) supplementation is becoming popular in shrimp feed formulation and is needed in low fishmeal diets as common alternative protein sources are typically deficient in essential amino acids, such as methionine and lysine. Furthermore, the slow feeding behaviour of shrimp compared to other cultured aquatic species means that feeds can lose specific nutrients before they are consumed. In particular, highly soluble CAA are prone to leaching. In this study, we examined the feeding behaviour, CAA leaching loss and AA uptake in large *Penaeus monodon* juveniles through a series of short-term experiments using a terrestrial meal-based formulation (TM) enriched with CAA and a traditional fishmeal-based formulation (FM). Feeding behaviour and nutrient bioavailability was found to be similar for the two diets. However, leaching over as little as 60 min had a major impact on AA absorption for the TM diet. The growth implications associated with leaching losses need to be investigated. However, the results indicate the need for careful feeding management as increased reliance on CAA for *P. monodon* culture could lead to suboptimal nutrition.

**Abstract:**

The complete replacement of fishmeal with terrestrial meals did not have a negative impact on the attractiveness, palatability, and apparent digestibility of the formulation. Shrimp were found on average to eat more and have similar appetite revival on the terrestrial meal-based formulation (TM) diet compared to the traditional fishmeal-based formulation (FM) diet. However, methionine (Met) and lysine (Lys) leached out rapidly from the TM diet, and as a result, this initially overfortified diet showed lower levels of those AA in comparison to FM after 60 min immersion. Both dietary Lys and Met were sub-optimal in TM within 120 min of immersion, whereas in comparison, the FM diet supplied consistent levels of EAA for up to 240 min immersion. Nonetheless, shrimp fed fresh TM had significantly higher peak haemolymph concentrations at 30 and 60 min for total AA, Met, and Lys than FM-fed shrimp. The over-supply of CAA far compensated leaching losses, and CAA were well absorbed and used by the shrimp within 120 min, with no obvious signs of asynchronous absorption of CAA to protein-bound AA. However, shrimp fed the TM diet that had leached out for 60 min, had haemolymph concentrations of Met and Lys that were only 41% and 44% of the ones on fresh feed respectively, while there was a negligible effect of leaching on FM. This study provides further insight into the feeding behaviour and bioavailability of dietary amino acids for *P. monodon* juveniles.

## 1. Introduction

Fishmeal or the use of wild-caught seafood remains an important part of today’s modern shrimp feed formulations, particularly for *Penaeus monodon*. Replacing fishmeal, and other wild-caught marine meals (e.g., krill, squid), with more sustainably sourced proteins in shrimp feed is needed on a global scale. Most studies have indicated an adverse impact on growth performance in clear-water laboratory systems when fishmeal is replaced completely by a selection of plant proteins (e.g., soybean, canola, rapeseed, mustard, peanut, rice, pea meals), rendered animal by-products (e.g., poultry, meat and bone) or insect meal [1,2,3,4,5]. These studies indicate that a small amount of fishmeal (10–15%) is generally required by *Litopenaeus vannamei* for optimum growth, survival or health. *P. monodon* appears to need somewhat higher inclusions (15–25%) for optimum performance [6], which can be offset using other marine ingredients (e.g., squid meal, krill meal or microbial biomass) [3,7,8]. Research is often conducted in laboratory conditions rather than field conditions, the latter benefiting from significant natural productivity, which can supplement an animal’s nutrition [9]. In green water systems and ponds, complete fishmeal replacement has been successfully demonstrated for *L. vannamei* [10,11], but *P. monodon* still showed lower growth performance when fed diets with ≤20% fishmeal [6].

With a growing trend towards the replacement of fishmeal in aquafeeds, crystalline amino acid (CAA) supplementation is becoming popular in shrimp feed formulation [12]. Dietary supplementation of CAA is not as widespread as in livestock and fish, and essential amino acid (EAA) requirements are instead met by increasing feed protein which can lead to the release of excess nitrogenous waste [13]. The slower feeding behaviour of shrimp compared to fish means that feeds can lose specific nutrients before they are consumed. This limits the ability to replace fishmeal by ingredients that require supplementation of essential CAA, such as methionine (Met), lysine (Lys), threonine (Thr), and arginine (Arg) [13] as well as the potency of functional water-soluble ingredients. Leaching losses end up shifting the formulated amino acid balance away from the requirements of the animal, and as a result shrimp can end up eating a lower quality diet.

Feed formulations can be designed to increase the water stability of pellets, but such steps may lead to a reduction in feed intake, as many water-soluble nutrients are known chemo-attractants and feed stimulants. Thus, if a diet is prepared in a way that reduces the solubility of these ingredients then it is possible that feed intake will decline. The effect of fishmeal replacement on diet attraction, palatability and the bioavailability of nutrients from other ingredient sources also needs to be understood [13]. Practically, leaching impacts are likely to be reduced by feeding multiple times a day and a clear benefit has been demonstrated in various recent studies for *L. vannamei* [13,14] and *P. monodon* [15]. Whilst these studies are useful and practical, there remains a lack of more fundamental investigations in characterising the effect of leaching on feeding behaviour and bioavailability of amino acids such as CAA in shrimp, particularly for a species like *P. monodon*. Two recently published studies have investigated the rapid absorption rate of amino acids from diets differing in test ingredients in *P. monodon* [16] and the use of different Met sources in *L. vannamei* [17], but the effect of leaching losses was not characterised.

In this study, we investigated the changes in composition through immersion of an experimental ‘zero fishmeal’ diet based on commercially available terrestrial ingredients (TM) which was supplied with CAA Met (1%), Lys (1%), and taurine (Tau, 0.3%) to exceed requirements. These changes were compared to a diet of comparable nutrient profile but composed of mainly fishmeal as the protein source (FM) with no added CAA. The specific aim of this study was to characterise the feeding behaviour (feed intake rate, appetite), digestibility and haemolymph amino acid uptake on those two diets when subject to variable periods of immersion (leaching time and feed deliveries). We examined the relationship between intake, amino acid leaching loss and amino acid uptake in shrimp through a series of short-term experiments and provide some recommendations for the feed management of shrimp in experimental and culture conditions, particularly on modern formulations requiring added CAA.

## 2. Materials and Methods

### 2.1. Diet Formulation, Composition and Macronutrient Digestibility

Two model experimental diets with similar proximate composition (i.e., 47–49% CP as is) and energy (i.e., 20.2 KJ g^−1^ as is) content were formulated with and without fishmeal (designated FM for fishmeal and TM for terrestrial meal respectively). Those protein and energy levels were above requirements for *P. monodon* but similar to other diets fed to *P. monodon* [8,18,19]. For the diet without fishmeal, poultry-by-product meal and plant (mainly soybean) derived meals were used. Wheat flour was reduced, and fish oil increased, to achieve similar lipid, carbohydrate and essential fatty acids to the FM diet (Table 1). In addition, this diet was fortified with CAA at the rate of 1% DL- Met, 1% L- Lys and 0.3% Tau (Table 1). Those inclusion levels were selected to ensure a small over-supply of all amino acids compared to the FM diet. The higher levels were used as it was envisioned that losses of CAA would occur through leaching.

Ingredients were milled to <500 µm before batching, and mixed in a Hobart mixer with water and oil added last to make up a dough of approximately 30% moisture content which was screw-pressed (Dolly, La Monferrina, Castell’Alfero, Italy) through a 3 mm die. Pellets were then steamed for 3 min and oven dried at 65 °C for 24 h. Diets were stored at −20 °C until required.

Yttrium oxide (3 µm particle size) was used as the indirect marker for apparent digestibility estimates [20]. Faeces were collected for two weeks from 6 communal tanks (100 L) containing 5 shrimp (mean weight = 24 g) fed either diet (*n* = 3). During this period, shrimp were fed twice daily, once at 0800 h for 1 h, the leftover feed removed from the tanks, and the faeces collected from the tank bottom every hour over three consecutive sampling occasions. Animals were also fed once at 1600 h to satiation allowing shrimp to feed overnight and tanks were siphoned cleaned of uneaten feed and faeces prior to the morning feed. Faeces were collected on a 250 µm screen, rinsed briefly with freshwater and pooled into their tank respective vials that were kept at −80 °C [8,20]. Apparent digestibility for dry matter, crude protein, total lipid, and gross energy were calculated as:

$$\begin{array}{c} Apparent\;digestibility\;coefficient\;\left(AD;\%\right)\\ \;=100\times \left[1-\left(\frac{Yttrium\;in\;the\;diet\;\left(Yd\right)}{Yttrium\;in\;the\;faeces\;\left(Yf\right)}\right)\times \left(\frac{Nutrient\;in\;faeces}{Nutrient\;in\;diet}\right)\right] \end{array}$$

### 2.2. Feeding Behaviour Experiments

Large *P. monodon* juveniles (15–25 g) were held individually in 60 transparent 6 L containers (0.26 m(L) × 0.15 m(W) × 0.18 m(D)) over the duration of the various short-term experiments. The containers received flow-through filtered seawater at a constant flow rate of 0.6 L min^−1^. Water temperature was maintained at 30 °C. High water quality was maintained throughout the experiment due to the high exchange rate (6 exchanges per hour). The entire system was illuminated at low levels with dimmable red SMD 5050 LED strips (wavelength = 625–660 nm) and photoperiod was kept at LD 24:0 h to eliminate differences in diurnal and nocturnal feeding activities. Two communal tanks containing animals fed either diet throughout the experimental period were used to re-stock the individual tanks. Intake or samples were not taken from animals 24 h before and after a moult, as moulting is known to affect feeding behaviour.

#### 2.2.1. Feed Intake Calculation

For the various experiments, feed intake was measured by delivering an amount equivalent to a 5% BW, which was considered sufficient to exceed satiation. The animals were then left to feed for the specified period after which left over feed was measured by siphoning on a fine screen (250 µm), rinsing thoroughly with deionized water, and drying at 105 °C for 4 h in individual pre-weighed aluminum trays. To enable sampling at exact time points, feeding times were staggered between the various tasks. In this regard, the introduction of food to the water was designated as 0 h for all experiments.

Dry matter content was measured by drying samples in an oven at 105 °C and all feed intake data is expressed on a dry matter basis. A water stability assessment was performed on the two diets in which 1 g of feed was added to tanks without animals, with each diet and feed combination repeated four times, and recovered after different time periods by siphoning through a 250 µm screen, as per the normal feed recovery procedure. Feed intake was calculated using the formulae outlined below:

$$Ind.\;feed\;intake\;FI\;\left(g\;DM\right)=Feed\;IN\;\times \;DM\;-\;\frac{Feed\;OUT}{WS}\times 100$$

$$Specific\;feed\;intake\;SFI\;\left(\%\;BW\right)=\frac{FI}{animal\;wet\;weight\;}\times \;100$$

where *Feed IN* is the amount of feed delivered as is, *DM* is the dry matter content of the feed, *Feed OUT* is the amount of dry matter recovered from each tank, and *WS* is water stability percent of each feed.

#### 2.2.2. Feed Intake and Appetite Revival after Single and Multiple Deliveries of Food

The rate of feed intake was measured by recording intake of shrimp that had been previously fasted overnight (for 16 h) at different periods following a single feeding event. Fasting was applied to ensure shrimp had empty guts and reached basal metabolic levels prior feeding [16,17]. Five replicate tanks for each combination of time (10, 30, 60, 90, 120, 240 min) and diet (FM and TM) were used per day, and the experiment repeated three times to achieve up to *n* = 15 replication per time point. Shrimp were assigned to the same feed treatment throughout the experiment but were randomly assigned a new experimental time treatment after a 24 h feed recovery.

The effect of multiple feed delivery on feed intake over 360 min was investigated by feeding shrimp once (time 0), twice (time 0 and 180 min) and three times (time 0, 120, 240 min). Overall feeding ration (equivalent to 5% BW) remained the same regardless of number of feed deliveries (i.e., rations divided by 50% and 33% for the 2 and 3 deliveries, respectively). For these experiments, remaining feed was only collected after the final period, so as not to unduly disturb the animals.

Appetite revival (AR) was determined as per [21] as follows: 16 h fasted shrimp were initially fed a meal (meal 1) equivalent to around a 1% BW ration (0.15–0.25 g) over a period of 60 min. If the shrimp was able to completely consume all the food within this time, it was then used for the appetite revival study. For this, five replicate shrimp were then fed a second meal at one of 60, 120, 180, 240, 360, or 500 min for the two diets. The experiment was conducted four times to achieve up to *n* = 20 replication per time point. Shrimp were randomly assigned a new experimental time treatment after a 24 h fed recovery period, whereas the two feed treatments remained assigned to the same animals throughout.

#### 2.2.3. Feed Intake Response on Leached out Feeds

The effect of leaching on feed intake was examined by soaking pre-weighed individual rations (equivalent to 2% BW) in a mesh-covered receptacle located in each aquarium that housed the experimental shrimp. Soaking periods were: 0, 30, 60, 120, 240 min. After soaking, the feed was made available to the shrimp that had previously been fasted for 16 h. This was achieved by slowly removing the receptacle containing the feed. The amount of feed remaining after 60 min of feed availability was then collected, dried and weighed. Five replicate tanks for each period of soaking and diet were used per day, and the experiment conducted three times to achieve up to *n* = 15 replication. Shrimp were randomly assigned a new experimental soaked treatment after a 24 h fed recovery period, whereas the two feed treatments remained assigned to the same animals throughout. Appropriate water stability controls were obtained by performing the same feeding and feed recovery procedure in tanks without animals.

### 2.3. Quantifying Nutrient Losses through Feed Leaching

Water stability of diets on a dry matter basis was measured by recovering feed from empty tanks after 10, 30, 60, 120 and 240 min, with each time point repeated 4 times. Protein and amino acid composition were measured in triplicate after recovering feed from various immersion periods in a horizontal orbital shaker (200 rpm) at 10, 30, 60, 120 and 240 min following a modified method from [22]. A 10 g sample of feed was soaked in 200 mL of seawater at 30 °C and agitated to allow constant water movement. All samples of pellets were recovered through a 250 µm screen, rinsed briefly with deionized water, dried at 105 °C for 4 h, and kept in individual jars for further analyses.

### 2.4. Haemolymph Amino Acids

Haemolymph was sampled at the completion of the feed intake experiments. Shrimp fed their respective diets for several weeks of feed intake measurement were fasted for 16 h prior to being fed a pre-weighed ration equivalent to 1% BW (e.g., 0.2 g for a 20 g shrimp). The shrimp were euthanised on ice after a period of 0 (unfed), 30, 60 or 120 min. A 1% BW ration was the maximum shrimp were able to eat within the 30 min time period, as per the result of the feed intake experiments. Only shrimp that were unfed (basal status, time 0) or observed to eat all their allocated ration within 30 min were used. Each combination of diet and period was sampled five times. Shrimp were also fed for 60 min with soaked feed pellets that were immersed for 30 and 60 min, as per Section 2.2.3. Haemolymph sampling was carried out as per [23]. The pericardial cavity between the cephalothorax and the first abdominal segment was punctured using a 25-gauge needle, and the haemolymph collected using a 200 μL pipette and diluted 1:2 with cold shrimp saline solution (SSS; 10 mM HEPES, 450 mM NaCl, 10 mM KCl, 10 mM EDTA) in a pre-weighed 1.5 mL centrifuge tube so that accurate haemolymph dilutions could be calculated. These samples were then centrifuged at 5000× *g* for 5 min at 4 °C immediately after sampling. The supernatant (plasma) was transferred to a new 1.5 mL centrifuge tube and immediately frozen on dry ice before storage at −80 °C.

The method to measure free amino acids in the haemolymph was derived from [24]. Briefly 50 µL of plasma was diluted with an equal volume of internal standard (500 µM α-amino butyric acid) solution. Proteins were then precipitated by adding acetonitrile (400 µL) and then spinning the samples at 2200× *g* for 4 min at 4 °C. The supernatant was then removed and a portion derivatised and analysed in the same manner as the feeds (see Section 2.5).

### 2.5. Chemical Composition Analyses

Feed samples (as is and leached out), as well as faeces for apparent digestibility, were analysed for chemical composition. Dry matter content was determined by gravimetric analysis following drying at 105 °C for 6 h. Ash content was determined based on mass change after combustion in a muffle furnace at 550 °C for 6 h. Total lipid content was determined gravimetrically following extraction in 2:1:0.4 chloroform:methanol: water, using a modification to the method proposed by [25]. A laboratory reference material consisting of a well-characterised diet was analysed concurrently for quality control. Nitrogen content was determined by the Dumas method using a Flash 2100 Elemental Analyser (Thermo Fisher Scientific., Waltham, MA, USA) and used to calculate sample protein content based on N × 6.25. The certified reference materials glutamic acid and 2-(N-morpholino) ethanesulfonic acid were used. Gross energy was determined by isoperibolic bomb calorimetry in a Parr 6200 oxygen bomb calorimeter with an 1108CL bomb for ingredients and diets, and an 1109A semi-micro bomb for faeces (Par Instrument Company, Moline, IL, USA). Benzoic acid was used as a reference. Carbohydrate was calculated by difference. For all proximate analyses, appropriate QC was undertaken and LOQ was 1 g kg^−1^ DM for all analyses, except for 1.1 KJ g^−1^ for energy.

Amino acid quantification was performed by mass detection following high performance reverse-phase liquid chromatography with pre-column derivatisation with 6-aminoquinolyl-N-hydroxysuccinimidyl (AQC). Analyses were undertaken on a Shimadzu Nexera X2 series UHPLC (Shimadzu Corporation, Kyoto, Japan), coupled with a Shimadzu 8030 Mass Spectrometer using a modification of the Waters AccQ-tag system (Waters Corporation, Milford, MA, USA). Samples of feed, including Bovine Serum Albumin (BSA) as reference standard, were hydrolysed using phenolic 6 N HCl at 112 °C according to the protocol for complex feed samples outlined by Waters Corp (1996). Cysteine and methionine can be lost in the hydrolysis and so were measured in separate assays whereby samples were initially oxidised with performic acid, which converts these compounds to cysteic acid and methionine sulfone respectively [26].

### 2.6. Statistical Analyses

Differences in dietary treatments were tested by one-way ANOVAs or two-way ANOVAs with time and diet as factors where possible, and post-hoc comparisons using Tukey-Kramer tests. Before all analyses, the ANOVA assumptions of normality of residuals and homogeneity of variances were tested using the Shapiro–Wilk and Levene tests, respectively. All analyses, unless otherwise indicated, were performed using NCSS 11.

## 3. Results

### 3.1. Feed Intake and Appetite Revival after Single and Multiple Deliveries of Food

**Figure 1:**
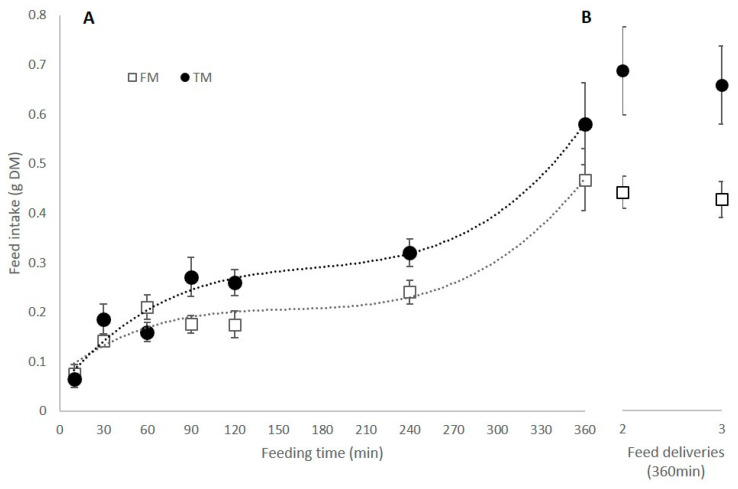
(**A**) Mean feed intake of individual shrimp on a single feed delivery over various feeding durations; (**B**) Mean feed intake over a 360 min feeding duration when fed twice (at time 0 and 180 min) and three times (0, 120 and 240 min). FM: traditional fishmeal-based formulation; TM: terrestrial meal-based formulation.

Feed intake increased with feeding duration (F = 31.64, *p* < 0.001) and was significantly higher for the TM diet than the FM diet (F = 6.99, *p* < 0.01). Feed intake within 10 min was significantly lower, and in 360 min significantly higher, than for all other time periods (Tukey-Kramer, *p* < 0.05). The response was, however, not linear with animals tending to reach a feed intake plateau between 60 min and 240 min (*p* > 0.05), and then increasing their intake further by 360 min (Figure 1A).

Feeding the same ration multiple (once, twice, or three) times had no significant effect on overall intake over the 360 min time period for each of the two diets (F = 0.17, *p* > 0.05) (Figure 1B). Overall intake of the TM diet was again higher than the FM diet (F = 12.8, *p* < 0.01) (Figure 1B).

**Figure 2:**
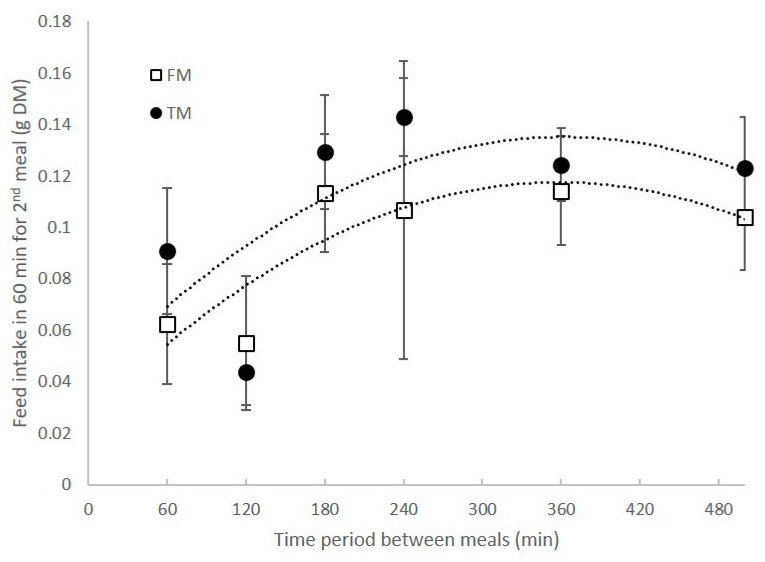
Revival of appetite in shrimp, expressed as the amount of feed consumed in the second meal, with different periods between meals.

Appetite revival, expressed as the amount of food eaten in a second meal, was significantly affected by the period between meals (F = 4.29, *p* < 0.01). Appetite was found to increase significantly when shrimp were allowed 180 min between the start of the two meals, compared to those fed consecutive meals within 120 min. There was no significant effect of diet on appetite revival (Figure 2).

**Figure 3:**
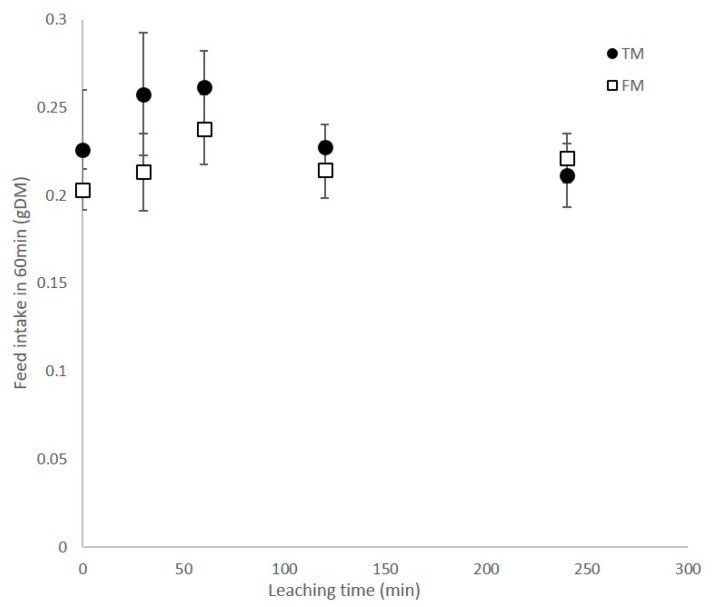
Feed intake over 60 min for shrimp fed diets that had been leached in situ for various times prior to feeding.

No significant difference in feed intake was observed between the two diets subjected to various immersion times (Figure 3; F = 0.95, *p* > 0.05). A consistent trend for a higher feed intake on TM was observed in this experiment but the response was not significant.

### 3.2. Feed Apparent Digestibility

The TM diet was as well digested by the shrimp as the FM diet for all macronutrients. No significant difference in apparent digestibility between the two diets was found (Table 2).

### 3.3. Feed stability and Leaching of Protein and Amino Acids

The two diets differed in water stability through time, with the FM diet being more stable after 120 min than the TM diet (two-way ANOVA, interaction, F = 3.11, *p* < 0.05) (Table 3). Both diets showed good water stability overall (>87% in 240 min). Crude protein content on a dry weight basis of the recovered feed did not vary significantly between diet and immersion time (TM: 460–518 g kg^−1^; FM: 470–498 g kg^−1^) (Table 3).

**Figure 4:**
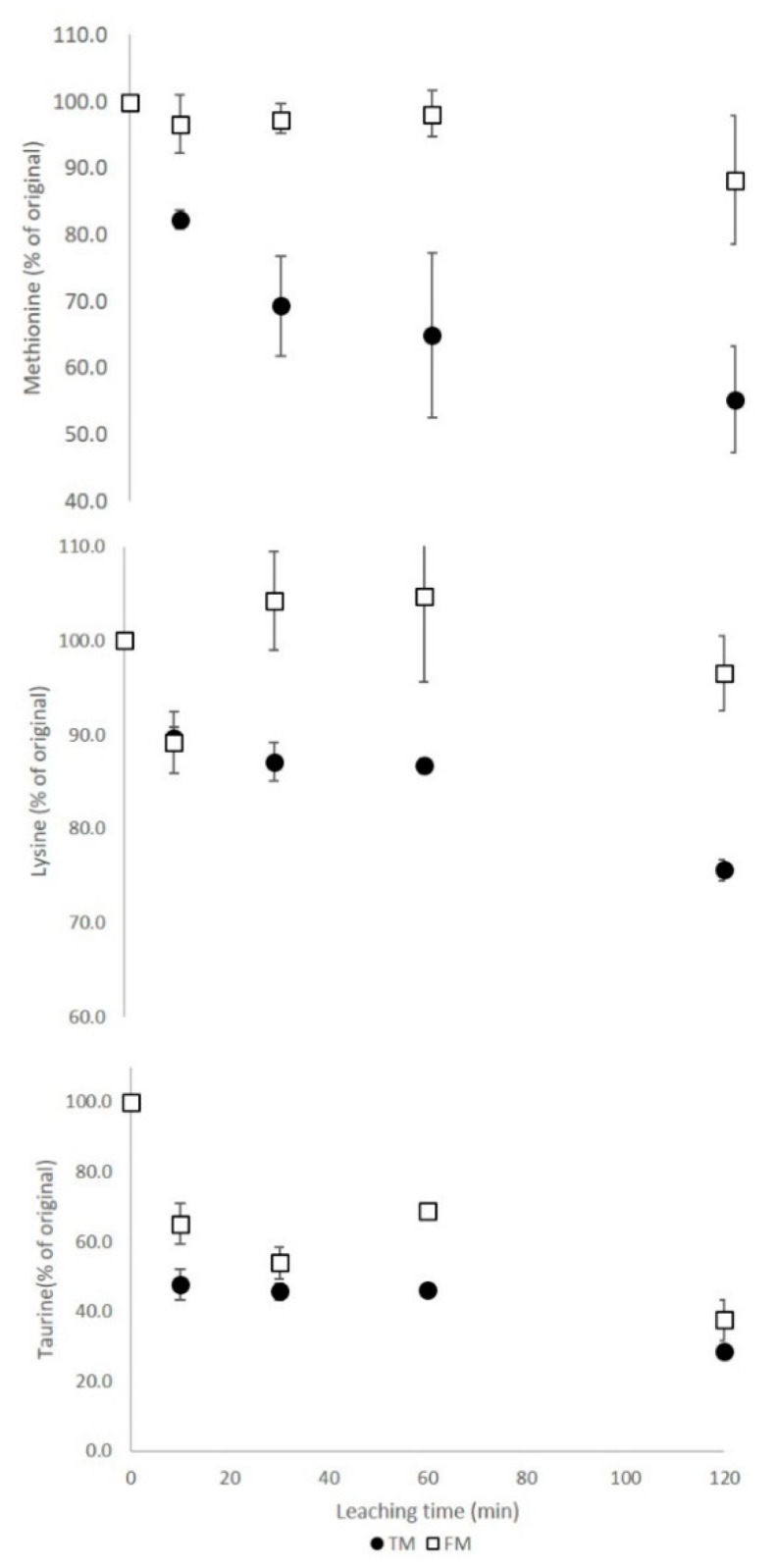
Methionine, lysine and taurine content, expressed in proportion of original, of two diets subject to leaching for various duration. Note the rapid drop of each amino acid from time 0 to the first sampling time (10 min), particularly for the TM diet.

There were some significant differences in the amino acid profile of the two diets. The TM diet contained significantly more arginine (+30%), aspartic acid (+13%), cysteine (+50%), glutamic acid (+25%), phenylalanine (+20%), and proline (+32%), while the FM diet contained more alanine (+11%) on average over the various immersion times (Table 3). Immersion time did not have a significant effect on amino acid composition (expressed in absolute amounts) of the two diets, except for Met (Table 3). When expressed in proportion of the original formulations, significant difference in Met (diet, F = 28.9, *p* < 0.001; time, F = 3.88, *p* < 0.05), Lys (interaction, F = 2.7, *p* < 0.05) and Tau (diet, F = 32.2, *p* < 0.001; time, F = 85.15, *p* < 0.001) were found for the TM diet (Figure 4). These three amino acids were added in crystalline form to TM but not FM. The leaching profile of the other amino acids was not significantly different between diets.

### 3.4. Haemolymph Amino Acids

**Figure 5:**
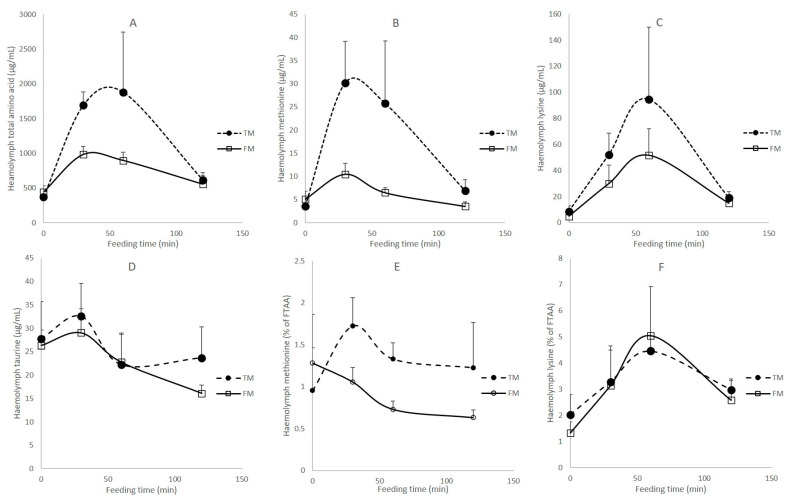
Haemolymph amino acid (µg mL^−1^) in shrimp fed two diets over time (min). (**A**) Free total amino acid (TAA) concentration; (**B**) free methionine concentration; (**C**) free lysine concentration; (**D**) taurine concentration; (**E**) free methionine in proportion to TAA (%); (**F**) free lysine in proportion to TAA (%). Note, all shrimp were given the same ration in terms of percentage body weight.

Rapid changes in haemolymph amino acid concentrations occurred following feeding. These changes occurred for both diets, with AA concentrations peaking between 30 min and 60 min after the introduction of feed and returned to pre-feeding levels by 120 min (Figure 5). Individual variation in haemolymph amino acid concentrations was high so many changes were not significant despite the use of 5 replicates per time point. Nonetheless, time of sampling post-feeding had a significant effect on concentrations of free EAA, NEAA, and all free individual amino acids (two-way ANOVAS, *p* < 0.05), apart from His, Arg, Asp, Glu, Tau (*p* > 0.05). Higher concentrations of free total amino acids (TAA), EAA, NEAA, and Met, Ile, Phe, Pro were found in the haemolymph of shrimp fed TM compared to FM (two-way ANOVA, *p* < 0.05) (Figure 5A,B shows the trends for TAA and Met). No significant difference between diets was found for haemolymph Lys (Figure 5C), Tau (Figure 5D) and other free amino acids. Met, in proportion of total free amino acid in the haemolymph, was significantly higher on TM post-feeding compared to FM (two-way ANOVA, *p* < 0.05) (Figure 5E) but Lys had a similar profile for both diets (Figure 5F).

**Figure 6:**
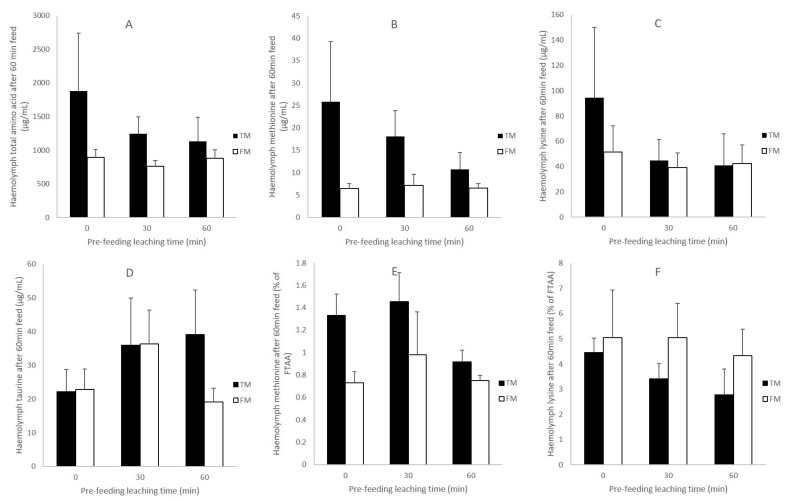
Haemolymph amino acid (µg mL^−1^) in shrimp fed for 60 min on diets that had been previously leached for 0 (fresh), 30 min and 60 min. (**A**) Free total amino acid (TAA); (**B**) free methionine; (**C**) free lysine; (**D**) taurine; (**E**) free methionine in proportion to TAA (%); (**F**) free lysine in proportion to TAA (%). Note, all shrimp were given the same ration in terms of percentage body weight (1% BW).

Soaking diets prior to feeding had a greater effect on haemolymph AA concentrations for shrimp fed the TM diet than occurred for those with the FM diet. For the TM diet, soaking for either 30 or 60 min prior to feeding led to lower levels of haemolymph amino acids after 60 min feeding compared to those fed on fresh feed. This was observed through reductions in TAA (Figure 6A), Met (Figure 6B) and Lys (Figure 6C). For shrimp fed the FM diet, similar haemolymph amino acid concentrations regardless of soaking time was found (Figure 6). Haemolymph taurine did not show a clear trend with leaching time for both diets.

Significantly higher haemolymph Met in proportion to TAA, was found in shrimp fed fresh TM compared to FM (one-way ANOVA, *p* < 0.05), but not when feeding on a 30 or 60 min leached feed (one-way ANOVA, *p* > 0.05) (Figure 6E). Similarly, a trend for a decreasing proportion of haemolymph Lys to TAA was found for shrimp fed TM subject to increasing leaching time (i.e., a 38% decrease between fresh and 60 min), while the trend was less evident for FM (i.e., a 14% decrease between fresh and 60 min) (Figure 6F).

## 4. Discussion

Complete replacement of fishmeal by quality terrestrial ingredients including soy products and poultry-by-product meal did not affect the attractiveness, palatability and digestibility of the formulation in *P. monodon* juveniles. In fact, shrimp were found on average to display higher feed intake rate, eat more over 6 h, and have similar appetite revival rates on the TM diet compared with those on the FM diet. In addition, the apparent digestibility of macronutrients in TM was equally as good as the fishmeal-rich diet. This was somewhat surprising considering that diets with FM substituted with plant proteins are often considered inferior for *P. monodon* and that inferiority was perceived to be caused by lower digestibility and/or palatability of these ingredients [7]. While the TM diet showed poorer water stability through time and greater losses of amino acids due to leaching losses, this did not affect its attractiveness over several hours of immersion. Shrimp sustained the same dry matter intake on feeds soaked for up to 240 min as on fresh food and ate an equal amount in DM regardless of the feed delivery frequency (once, twice or three times over 360 min). It is worth noting that in our experiments, shrimp were fasted for 16 h before being fed, so it is possible that more subtle differences in attractiveness between the two diets would be noticeable if shrimp were fed under more constant feeding conditions. However, our results concur with previous studies using diets with no, low or high amounts of fishmeal [3]. Together, these findings indicate that the reduced growth observed to date on diets with no fishmeal for *P. monodon*, as found in [3,6,7], may not be linked to gross issues with feed intake or digestibility.

Leaching of crystalline AAs reduced the levels of these key nutrients through time. The TM diet in this study was pre-emptively over-fortified to help counter this issue. However, within 60 min the total Met and Lys content of TM ended up below that of the fishmeal-based diet. In contrast, the FM formulation, which had no additional CAA, had a stable protein and amino acid composition through immersion of up to 240 min, apart from Tau which decreased to 37% of the original amount within 120 min. These results are consistent with previous studies on leaching from aquaculture feeds, with Tau occurring naturally in free form rather than bound within proteins [27,28]. The leaching of CAA made a diet, once over-fortified for Met (14 g kg^−1^), become Met deficient (8 g kg^−1^) after 120 min immersion for *P. monodon* [6]. Expressed in percentage of crude protein, the TM diet after 60 min leaching contained 1.9% Met, which is below the requirement reported previously of 2.4% for maximum growth and 2.9% for maximum N gain [29,30]. Total sulphur amino acids (Met + Cys) levels were just satisfying requirements at 60 min [30]. Lys in TM also showed similar trends, with levels dropping to 4.8% of crude protein by 120 min while FM maintained levels >5.5% which is above requirement of 5.2% for growth [31] and close to maximum N gain requirement of 5.8% [30].

Shrimp continued to feed on the TM diet even after the level of crystalline AAs had declined to low levels through leaching. This continued feeding may be of concern to the aquaculture industry, as it indicates that shrimp are likely to become satiated on feeds that are providing sub-optimal nutrition. Shrimp ate these diets for up to 360 min. They also showed no clear reduction in feed intake over a 60 min period when fed feed that had been previously soaked for up to 240 min. The ability of shrimp to discern nutritional qualities of fresh vs. soaked feed, and their capacity to shift consumption to fresh food, was not tested in this study but would be worthy of investigation. However, no improvement in feed intake was observed when feed was delivered more frequently, being once, twice or three times over 6 h. These finding indicates that having excess food delivered could lead to nutritional losses when CAA or other water-soluble compounds are used in diets. A management response may be to prescribe a high delivery frequency of small amounts of feed. Such a regime would enable food to be consumed within short periods and ensure that rations are of the highest quality.

Changes in haemolymph AAs were measured to characterise the effect of leaching on amino acid uptake and absorption and to provide insights into protein metabolism on those two diets. The observed AA fluxes in haemolymph in this study are hypothesised to have originated from the diet, rather than endogenous sources, due to the rapid rise in total amino acid concentration after feeding (within 30 min) from pre-fed levels [32]. The post-prandial haemolymph concentrations of TAA, Met, and Lys, reached higher levels and at a quicker rate in TM-fed, compared to the FM-fed shrimp. Amino acid concentrations were similar with recent studies, with TAA in 16 h unfed shrimp around 400 µg mL^−1^ and reaching post-prandial peaks as high as 2000 µg mL^−1^ within 60 min [16,17]. The higher levels and rapid increases for Met and Lys were likely due to the addition of CAA in the TM diet. CAAs do not require digestion and so are readily available for absorption into haemolymph as observed in several studies in aquatic animals [33,34,35]. We can attribute the higher levels of other haemolymph free amino acids in shrimp fed the TM diet to a slightly higher protein content, apparent protein digestibility and slight excess in most amino acids supplied in fresh feed (Table 3), as feed intake was kept the same as the FM diet (i.e., a restricted ration of 1% BW). Shrimp were able to readily utilise AA from both the TM and FM diets, as suggested by the subsequent drop in haemolymph concentrations of Met, Lys and total amino acids. Met has a relatively high turnover rate in cellular metabolism undergoing high rates of oxidation as a methyl and sulphur source [36] while Lys is one of the two most limiting amino acids and a substrate for muscle protein [37]. Shrimp fed either diet had haemolymph concentrations return to basal levels by 120 min post-prandial, despite the significantly higher peak in TM-fed shrimp at 30 min and 60 min. Furthermore, this return to basal levels appears to occur prior to the peak in appetite revival. Those results indicate a relatively similar digestive and absorptive process rate in *P. monodon* compared to *L. vannamei* [17]. When fed fresh food, there was no obvious issues with feeding TM despite the leaching losses in Met and Lys measured in as little as 10 min immersion, with the over-supply far compensating the leaching losses, and CAA being well absorbed by the shrimp. In addition, no obvious signs of asynchronous absorption of Met and Lys to the rest of dietary protein was found for TM, in agreement with [17], indicating very fast rates of digestion of protein bound amino acids as found in a recent study [16].

Leaching of the TM diet had a marked effect on the capacity for shrimp to absorb AA. Shrimp fed the TM diet that had been soaked for 60 min had haemolymph concentrations of Met and Lys that were only 41% and 44% of the ones on fresh feed respectively, while there was a negligible effect on shrimp fed FM under the same conditions (i.e., 2% increase in Met and 17% decrease in Lys compared to fresh). Feeding leached diets did not result in lower absolute concentrations of free Met and Lys in the haemolymph of TM-fed shrimp compared to FM-fed shrimp after 60 min feeding due to the overfortification of the former. However, in the case of TM Lys, a trend for a reduction in haemolymph Lys in proportion of total haemolymph free amino acid was evident with increasing pre-feeding leaching times. This suggests commercial formulations would require the overfortification of CAA beyond species requirements in order to deliver a suitable amount post-immersion, to account for leaching and the feeding behaviour of *P. monodon*. Those observations compare well to a recent study demonstrating lower growth performance of *L. vannamei* fed a 60 min soaked commercial diet [26]. Overfortification of Lys and Met is needed as a mitigation strategy, but this can be a big expense particularly in least-cost formulations and could result in an amino acid imbalance dependent on feed intake, as well as losses to the environment. In addition, most of the losses occurred in the first 10 min of immersion, which our data indicate is an insufficient period for shrimp to complete feeding.

Leaching of CAA in aquatic diets is a focus area in aquaculture nutrition due to the increased availability of these products [12]. Attempts to increase their stability in diets have been promising, such as the use of novel Met containing products like AQUAVI^®^ Met-Met [38], however the effect of coating a broader range of CAA and/or diet on their bioavailability remains unclear. For example, [39] showed coating CAA with a binder such as agar or carboxy-methyl cellulose was a useful technique to retard leaching losses in water and resulted in positive growth response in shrimp (*Marsupenaeus japonicus*). Diets containing uncoated CAA had greater leaching of total amino acids than diets containing coated or protein-bound amino acids. Other factors, such as attractiveness, palatability, and digestibility, are likely to also be negatively affected by coated CAA, so a compromise between water stability and release during digestion needs to be achieved which warrants future research.

Leaching of CAA from diets and the time taken for feed intake and appetite revival of large juvenile *P. monodon* have implications for aquaculture. To reduce losses from leaching, feed should only be delivered in small quantities at a time, ideally enabling consumption within 30 min. The frequency of feeding should in turn be dictated by how fast appetite is revived. This study demonstrates that appetite is highest when meals are separated by 180 min. Together these findings would indicate that a feeding frequency of eight times a day (every 180 min) would maximise intake rate and appetite. Obviously, those recommendations assume all shrimp have access to food as soon as delivered, which may not always be the case in commercial systems at high stocking densities. Further feed management research is warranted in larger scale system, subject to variation in diurnal feeding activities.

## 5. Conclusions

We examined the relationship between intake, amino acid leaching loss and amino acid uptake in shrimp through a series of short-term experiments to understand potential nutritional deficiencies of an experimental diet with no marine meal (TM) compared to a fishmeal-rich control diet (FM). We found no apparent issues with the attractiveness, palatability and apparent digestibility of the formulation. While the TM diet is less stable in water and can lead to sub-optimal levels of EAA within 120 min of immersion compared to FM, shrimp fed fresh feed showed no issues with AA absorption rate and use. However, if the feed is immersed prior to consumption, the effect of leaching losses becomes apparent with haemolymph concentrations of Met and Lys of less than half those on fresh feed. In contrast, the FM diet retained nutritional content over long immersion periods (240 min). As such, the issue with replacing fishmeal entirely in diets for *P. monodon* maybe associated with losses of crystalline amino acid (CAA) and poorer amino acid profile post-immersion, compounded by reduced access to fresh feed and/or insufficient feeding frequency. The growth implications of reduced AA absorption from these diets remains to be investigated. More research into minimising the leaching loss of water-soluble compounds and farm feeding management of low FM diets appear important to guarantee their commercial success with *P. monodon.*

## Figures and Tables

**Table 1:** Dietary treatment formulation, composition on DM basis and digestible basis.

g kg^−1^	FM	TM
Fishmeal	500	0
Poultry by-product meal	0	200
Full-fat soybean meal	0	250
Soy protein concentrate (SPC)	0	120
Gluten	70	100
Wheat flour	395	262
Fish oil	15	25
DL-Methionine (Met)	0	10
L-Lysine (Lys)	0	10
Taurine (Tau)	0	3
Soy lecithin	10	10
Cholesterol	1	1
Choline (60% choline chloride)	5	5
Vitamin premix	2	2
Vitamin C (stay C)	1	1
Yttrium oxide	1	1
Astaxanthin	0.5	0.5
Banox E	0.2	0.2
**Feed Proximate Composition (on Dry Matter Basis, except for Moisture as Is)**		
Moisture (%)	3.9	5.0
Crude protein (CP) (%)	49.8	51.8
Gross energy (KJ g^−1^)	21.0	21.3
Total lipid (%)	8.7	7.6
Ash (%)	8.3	6.2
Carbohydrate (%)	33.1	34.4

Fishmeal, poultry-by product meal, fish Oil: Ridley, Narangba, QLD, Australia. Wheat flour and wheat gluten: Manildra, Auburn, NSW, Australia. Soybean meal: Kewpie Stockfeeds Pty Ltd., Kingaroy, QLD, Australia. Soy protein concentrate (SPC): Selecta, Jardim Goiás, GO, Brazil.

**Table 2:** Apparent digestibility of dry matter, crude protein, gross energy and total lipid for the two diets of this study. Results are mean ± S.E. (*n* = 3). No statistical difference was found.

Apparent Digestibility (%)	FM	TM
Dry matter	63.2 ± 6.9	65.3 ± 4.0
Crude protein	77.7 ± 3.8	82.7 ± 0.1
Gross energy	82.9 ± 3.1	80.5 ± 2.1
Total lipid	77.2 ± 3.7	77.7 ± 2.9

**Table 3:** Dry matter (DM), crude protein (CP), essential (EAA) and non-essential (NEAA) amino acid composition of two diets subject to various leaching periods in seawater. Data are means of three replicates and significant differences (*p* < 0.05 *, *p* < 0.01 **, *p* < 0.001 ***) between diets and periods are noted (two-way ANOVA, *p* < 0.05). Data in g kg^−1^ DM basis unless otherwise stated.

Diet	FM						TM						Stats	
Leaching Time (min)	0	10	30	60	120	240	0	10	30	60	120	240	Diet	Time
Water stability (% DM)	100	98.8	97.3	96.6	94.6	93.3	100	97.0	95.6	95.1	90.2	87.4	F = 28.5 ***	F = 33.9 ***
													Interaction, F = 3.11 *	
CP	498	490	495	470	478	486	518	504	485	460	475	473		
Total AA	398	408	403	453	404	413	459	472	481	447	474	459		
Arg	22	23	23	25	21	22	30	27	30	29	31	28	F = 21.99 ***	
His	10	10	9	14	9	9	9	9	11	10	10	9		
Ile	18	18	19	19	18	19	19	20	20	19	20	19		
Leu	32	33	32	34	32	32	33	35	36	33	35	33		
Lys	28	25	29	29	27	27	30	27	26	26	23	22		
Met	11	10	10	10	9	10	14	11	10	9	8	7		F = 3.05 *
Phe	19	20	20	21	19	20	23	24	25	22	25	24	F = 26.8 ***	
Thr	18	19	18	23	19	20	18	19	19	19	19	18		
Val	15	16	15	17	15	16	16	17	17	16	17	16		
Sum EAA	165	166	168	183	164	167	180	184	185	173	178	171		
Ala	23	24	23	27	25	25	21	22	23	22	23	22	F = 7.29 **	
Asp	35	36	36	42	36	37	41	43	42	40	43	41	F = 5.49 **	
Cys	5	6	6	6	5	6	8	9	9	7	9	8	F = 15.7 ***	
Glu	83	87	84	86	85	85	102	109	110	101	110	104	F = 25.6 ***	
Gly	23	24	23	28	23	24	24	25	26	24	26	24		
Pro	26	27	27	28	26	27	34	36	38	33	37	34	F = 51.0 ***	
Ser	18	19	18	33	22	25	23	24	25	23	25	25		
Tau	3	2	2	2	1	1	4	2	2	2	1	1		
Tyr	9	10	10	10	10	10	11	12	12	11	13	11		
Sum NEAA	233	241	236	270	240	246	279	288	296	274	296	289		

## Data Availability

The data presented in this study are available on request from the corresponding author.
